# Supplementary material for: Impulse oscillometry system for assessing small airway dysfunction in pediatric bronchiolitis obliterans; association with conventional pulmonary function tests
Source: PLoS One. 2023 Feb 7;18(2):e0280309. doi: 10.1371/journal.pone.0280309 (PMC9904454; doi:10.1371/journal.pone.0280309)
Supplement: S1 Table — This table shows the minimal data set that was used in this study. (PDF) [file pone.0280309.s001.pdf]

Patient_Nc	Age_yr	gender	Lung_diagı	diagnosis1	diagnosis2	diagnosis3	R5
IOS0001	<5	M	None	malignanc	Leukemia	AML	8.957
IOS0002	5-10	M	None	malignanc	Leukemia	ALL	9.302
IOS0003	5-10	M	None	malignanc	Leukemia	ALL	9.302
IOS0004	5-10	M	None	malignanc	Leukemia	AML	5.664
IOS0005	5-10	M	None	malignanc	Leukemia	AML	9.584
IOS0006	5-10	M	None	n-malignaı	Hemoglob	Hemoglob	6.487
IOS0007	5-10	M	None	malignanc	Leukemia	ALL	7.972
IOS0008	5-10	M	None	malignanc	Leukemia	ALL	10.704
IOS0009	5-10	M	None	malignanc	Leukemia	ALL	5.034
IOS0010	5-10	F	None	malignanc	Leukemia	ALL	6.641
IOS0011	5-10	F	None	malignanc	Leukemia	ALL	11.678
IOS0012	5-10	F	None	malignanc	Leukemia	ALL	5.649
IOS0013	16-18	M	BO	malignanc	Leukemia	AML	3.266
IOS0014	11-15	F	BO	malignanc	MDS/MPN	MDS	11.964
IOS0015	11-15	F	BO	malignanc	MDS/MPN	MDS	13.455
IOS0016	5-10	M	BO	malignanc	Leukemia	ALL	11.139
IOS0017	5-10	M	BO	malignanc	Leukemia	ALL	8.226
IOS0018	5-10	M	BO	malignanc	Leukemia	ALL	9.049
IOS0019	5-10	M	BO	malignanc	Leukemia	ALL	8.306
IOS0020	5-10	M	BO	malignanc	Leukemia	ALL	9.868
IOS0021	5-10	M	BO	malignanc	Leukemia	ALL	9.796
IOS0022	5-10	M	BO	malignanc	Leukemia	ALL	10.214
IOS0023	5-10	M	BO	malignanc	Leukemia	ALL	6.735
IOS0024	11-15	M	BO	malignanc	Leukemia	ALL	4.019
IOS0025	11-15	M	BO	malignanc	Leukemia	ALL	3.59
IOS0026	11-15	M	BO	malignanc	Leukemia	ALL	4.711
IOS0027	11-15	M	BO	malignanc	Leukemia	ALL	3.867
IOS0028	11-15	M	BO	malignanc	Leukemia	ALL	3.695
IOS0029	11-15	M	BO	malignanc	Leukemia	ALL	3.892
IOS0030	11-15	M	BO	malignanc	Leukemia	ALL	3.936
IOS0031	11-15	M	BO	malignanc	Leukemia	ALL	4.153
IOS0032	11-15	M	BO	malignanc	Leukemia	ALL	3.737
IOS0033	11-15	F	BO	malignanc	MDS/MPN	MDS	3.492
IOS0034	11-15	F	BO	malignanc	Leukemia	AML	8.244
IOS0035	11-15	M	BO	malignanc	Lymphoma	Lymphoma	5.214
IOS0036	11-15	M	BO	malignanc	Lymphoma	Lymphoma	3.837
IOS0037	11-15	M	BO	malignanc	Lymphoma	Lymphoma	4.703
IOS0038	11-15	M	BO	malignanc	Lymphoma	Lymphoma	4.402
IOS0039	11-15	M	BO	malignanc	Lymphoma	Lymphoma	4.395
IOS0040	11-15	M	BO	malignanc	Lymphoma	Lymphoma	3.706
IOS0041	5-10	M	BO	malignanc	Leukemia	JMML	9.188
IOS0042	5-10	M	BO	malignanc	Leukemia	JMML	9.099

IOS0043	5-10	M	BO	malignancy	Leukemia	JMML	11.454
IOS0044	5-10	M	BO	malignancy	Leukemia	JMML	18.988
IOS0045	11-15	F	BO	BO, Asthm	BO, Asthm	BO, Asthm	4.117
IOS0046	11-15	M	BO	malignancy	Leukemia	ALL	10.658
IOS0047	11-15	M	BO	malignancy	Leukemia	ALL	10.483
IOS0048	11-15	M	BO	malignancy	Leukemia	ALL	7.344
IOS0049	11-15	M	BO	malignancy	Leukemia	ALL	8.188
IOS0050	16-18	M	BO	malignancy	Leukemia	ALL	6.669
IOS0051	16-18	M	BO	malignancy	Leukemia	ALL	5.541
IOS0052	16-18	M	BO	malignancy	Leukemia	ALL	7.779
IOS0053	16-18	M	BO	malignancy	Leukemia	ALL	7.368
IOS0054	16-18	M	BO	malignancy	Leukemia	AML	2.817
IOS0055	5-10	F	BO	malignancy	Leukemia	AML	10.45
IOS0056	5-10	M	None	n-malignan	Bonemarro	SAA	6.026
IOS0057	16-18	F	None	n-malignan	Bonemarro	SAA	3.437
IOS0058	16-18	M	BO	malignancy	Leukemia	ALL	9.105
IOS0059	11-15	M	BO	malignancy	Leukemia	AML	11.689
IOS0060	11-15	M	BO	malignancy	Leukemia	AML	13.039
IOS0061	11-15	M	BO	malignancy	Leukemia	AML	11.647
IOS0062	11-15	M	BO	malignancy	Leukemia	AML	10.538
IOS0063	11-15	M	BO	malignancy	Leukemia	AML	9.102
IOS0064	11-15	M	BO	malignancy	Leukemia	AML	7.736
IOS0065	11-15	M	BO	malignancy	Leukemia	AML	7.303
IOS0066	5-10	M	BO	malignancy	Leukemia	AML	6.823
IOS0067	11-15	M	BO	BO, Asthm	BO, Asthm	BO, Asthm	3.178
IOS0068	11-15	F	BO	malignancy	Leukemia	ALL	9.178
IOS0069	11-15	F	BO	malignancy	Leukemia	ALL	8.951
IOS0070	<5	M	BO	n-malignan	Bonemarro	SAA	7.669
IOS0071	5-10	F	None	malignancy	Leukemia	AML	9.484
IOS0072	5-10	M	BO	BO	BO	BO	11.9
IOS0073	5-10	M	BO	BO	BO	BO	7.51
IOS0074	5-10	M	BO	malignancy	Leukemia	ALL	8.015
IOS0075	5-10	M	BO	malignancy	Leukemia	ALL	7.473
IOS0076	5-10	M	BO	malignancy	Leukemia	ALL	6.023
IOS0077	5-10	M	BO	malignancy	Leukemia	ALL	7.816
IOS0078	<5	M	BO	BO, COP	BO, COP	BO, COP	13.005
IOS0079	<5	M	BO	BO, COP	BO, COP	BO, COP	13.362
IOS0080	<5	M	None	malignancy	Leukemia	CML	8.64
IOS0081	5-10	M	None	n-malignan	Bonemarro	SAA	10.167
IOS0082	5-10	M	BO	n-malignan	Hemoglob	Hemoglob	9.477
IOS0083	5-10	M	BO	n-malignan	Hemoglob	Hemoglob	11.955
IOS0084	5-10	M	SJS	BO, SJS	BO, SJS	BO, SJS	11.338
IOS0085	5-10	M	SJS	BO, SJS	BO, SJS	BO, SJS	9.637

IOS0086	5-10	M	SJS	BO, SJS	BO, SJS	BO, SJS	13.122
IOS0087	<5	M	PIBO	PIBO	PIBO	PIBO	13.18
IOS0088	<5	M	PIBO	PIBO	PIBO	PIBO	9.128
IOS0089	<5	M	PIBO	PIBO	PIBO	PIBO	12.997

R5-20	AX	X5	VT	COH5	R5_Zscore	AX_Zscore	R5_Pred
0.419	11.472	-0.07	0.401	0.932	0.7961	-1.314	119.4
2.201	26.274	-0.781	0.646	0.923	1.31	0.1578	133.9
2.201	26.274	-0.781	0.646	0.923	0.05265	-0.8042	101.2
-0.346	3.961	-0.676	0.305	0.969	-0.6193	-1.779	87.1
0.847	17.846	-0.403	0.401	0.913	1.739	-0.2342	147.4
1.476	34.578	0.381	0.559	0.878	-0.03059	0.8954	99.32
3.329	40.054	1.891	0.377	0.877	0.6868	1.044	116.6
3.446	70.399	-2.237	0.386	0.823	2.131	2.566	160.9
-0.128	11.794	0.554	0.43	0.879	-1.34	-0.931	74.17
1.129	23.296	0.758	0.198	0.856	-0.3503	-0.2502	92.49
2.256	59.512	-2.85	0.232	0.888	1.807	1.454	149.6
0.338	16.71	-3.211	0.157	0.936	-1.651	-1.212	69.19
0.1	5.322	-0.653	0.686	0.934	0.9577		130.8
6.703	191.934	7.43	0.462	0.899	3.863		330.4
8.381	186.482	8.766	0.52	0.866	4.243		371.6
6.384	174.049	4.697	0.452	0.932	4.84		447.1
4.058	140.76	4.991	0.48	0.935	3.86		330.2
4.081	97.754	1.574	0.481	0.926	4.168		363.2
4.102	95.595	1.429	0.456	0.947			
5.324	107.083	1.911	0.376	0.94	4.449		396.1
5.742	136.654	3.663	0.401	0.934	4.425		393.2
5.288	126.23	1.735	0.433	0.948	4.56		409.9
0.364	11.123	0.245	0.338	0.952	-0.1579	-1.096	96.54
1.817	37.143	-0.695	0.579	0.848	1.482	4.203	151.5
1.34	23.203	-1.016	0.566	0.873	1.079	3.556	135.3
2.354	33.481	-0.58	0.552	0.871	2.045	4.06	177.4
1.614	33.234	-0.708	0.552	0.876	1.341	4.05	145.6
1.6	31.233	-1.115	0.596	0.918	1.18	3.965	139.2
1.696	34.722	-0.905	0.617	0.889	1.366	4.11	146.6
1.766	31.152	-0.392	0.507	0.89	1.406	3.961	148.3
1.932	37.612	-0.591	0.641	0.88	1.598	4.221	156.5
1.467	29.742	-0.776	0.529	0.926	1.223	3.897	140.9
-0.209	4.424	-0.362	0.234	0.971	-0.2504		92.54
4.03	62.384	1.609	0.643	0.921	3.502	4.384	253.6
2.842	65.992	3.581	0.39	0.757	3.268	5.296	249.9
1.844	77.052	2.176	0.504	0.939	2.173	5.51	183.9
2.422	92.871	1.886	0.478	0.874	2.9	5.767	225.4
1.923	81.761	2.035	0.454	0.898	2.516	5.504	202.4
2.15	89.369	2.31	0.453	0.925	2.51	5.626	202.1
1.736	80.518	0.864	0.542	0.952	1.903	5.483	170.5
4.307	136.263	5.434	0.395	0.913	1.058	4.531	126.6
4.679	107.175	3.539	0.429	0.935	1.014	3.618	125.4

3.985	185.846	6.41	0.231	0.697	1.751	5.655	147.8
7.139	307.294	7.16	0.283	0.733	4.018	8.35	245
1.138	12.824	-0.43	0.36	0.945	-1.446	-0.2908	72.44
4.513	80.857	0.826	0.328	0.859	2.56	3.332	177
5.779	81.905	2.357	0.424	0.891	2.545	3.419	176.4
3.043	45.415	1.069	0.381	0.886	0.9492	1.821	123.6
3.997	59.293	2.807	0.401	0.902	1.437	2.485	137.8
3.708	63.097	1.268	1.049	0.954	2.683	4.538	212.1
2.736	37.853	0.843	0.96	0.945	2.022	3.835	176.3
4.445	77.2	2.143	1.33	0.921	3.232	4.816	247.4
4.146	75.439	2.204	1.179	0.932	3.039	4.784	234.4
0.439	5.433	-0.376	0.632	0.802	-0.1006		92.25
5.032	127.163	7.732	0.328	0.882	1.403	3.99	136.7
1.863	24.062	0.422	0.37	0.855	-0.0951	0.4293	97.9
-0.242	2.964	-0.235	0.584	0.949	1.077	0.7313	133.1
4.475	175.57	4.776	0.542	0.892	2.506	1.134	283.3
5.857	159.729	3.269	0.43	0.859	2.84	1.363	233.1
5.044	186.501	8.106	0.518	0.853	3.414	1.117	260.1
6.067	164.873	4.635	0.4	0.818	2.822	1.427	232.3
4.851	142.671	4.876	0.347	0.842	2.35	1.058	210.2
4.529	124.293	3.081	0.432	0.933	1.739	0.9607	181.5
3.988	133.421	3.764	0.548	0.931	1.158	0.7967	154.3
3.623	134	1.514	0.409	0.927	0.974	0.6862	145.7
-0.176	20.102	0.318	0.235	0.942	0.8311	0.4266	120.4
-0.299	1.973	-0.312	0.785	0.947	0.8588	0.1626	127.2
2.523	54.157	-0.027	0.562	0.968	1.369	0.3273	154
3.607	62.415	1.644	0.483	0.961	1.273	0.6557	150.2
-0.455	43.915	0.676	0.484	0.732	-0.3136	0.6024	93.25
0.398	17.101	-0.55	0.286	0.914	0.8692	-1.025	121.4
3	80.78	-0.251	0.492	0.906	2.769	3.106	185.4
0.535	14.344	-1.231	0.596	0.881	0.7047	-0.4903	117
0.608	8.861	0.4	0.281	0.952	0.05162	-1.791	101.2
1.185	8.821	-0.712	0.324	0.919	-0.2621	-1.795	94.32
0.789	12.987	-0.216	0.368	0.977	-1.229	-1.357	76.02
0.45	22.796	0.251	0.384	0.889	-0.06114	-0.5475	98.65
4.596	99.766	3.247	0.176	0.847	1.083	0.3254	124.3
4.222	176.386	2.549	0.184	0.854	1.235	0.2121	127.8
-1.64	68.134	-1.303	0.181	0.821	-0.1031	1.474	97.73
1.278	46.357	-0.128	0.268	0.97	1.364	1.052	135.6
-0.478	22.603	0.544	0.198	0.865	-0.1954	-1.145	95.38
0.181	27.287	-2.323	0.209	0.866	0.8589	-0.9456	120.3
5.245	69.484	-1.295	0.302	0.815	1.681	1.973	145.5
0.205	44.064	0.843	0.362	0.749	0.9519	0.799	123.6

5.056	139.418	-0.26	0.26	0.711	2.336	4.374	168.4
2.995	83.676	-0.492	0.332	0.892	2.05	2.21	157.9
1.309	33.296	-1.376	0.36	0.89	0.4022	-0.1256	109.4
3.879	50.483	-0.355	0.275	0.927	1.987	0.7975	155.7

AX_Pred	R5_CV	AX_CV	VT_CV	FVC	FEV1	FEV1_FVC	FEF25_75
41.03	3.871	9.066	8.746	82	84	90	128
109.5	11.75	32.04	21.34				
57.31	17.14	93.07	9.041	111	122	98	185
19.02	17.14	93.07	9.041				
85.71	17.14	93.07	9.041				
164.4	17.14	93.07	9.041	83	77	83	62
172.4	6.529	34.15	16.01	109	108	89	119
321.4	11.95	34.81	8.065	97	99	92	106
51.61	26.55	29.67	16.93	98	93	84	80
86.54	12.16	31.48	23.13	67	69	95	136
189	11	39.02	13.85	79	85	97	135
	6.835	29.66	33.75	73	81	97	124
277.3	0.3525	0.4239	0.03711	69	69	92	71
	2.066	10.86	0.0434	63	18	26	6
	0.9558	3.151	0.01574	33	11	33	5
	15.47	4.901	11.1				
	3.812	7.132	6.252				
	12.25	12.23	7.786				
	30.62	47.83	7.501				
	19.17	39.92	5.549				
	3.431	7.951	2.22				
	7.986	12.18	2.765				
45.96							
2120	8.973	2.678	8.002	61	45	68	14
1324	12.73	3.866	8.616				
1911	7.136	5.326	4.846				
1897	11.8	1.683	2.395				
1783	10.46	7.048	1.013				
1982	7.491	3.864	3.131				
1778	7.291	3.102	1.195				
2147	8.473	2.22	0.7967				
1698	4.589	5.681	11.76	62	46	70	15
	7.313	5.634	11.14	80	65	74	40
2098	8.903	2.727	3.738	63	33	45	10
4691	15.1	17.11	15.65				
5478	5.49	2.628	6.078				
6602	10.03	1.66	3.757				
5454	7.883	4.641	8.229				
5961	2.484	3.67	5.123				
5371	5.802	6.974	5.482				
519.7	17.25	13.77	7.835	48	25	60	12
408.8	5.763	0.7885	0.982				

624.6	5.943	16.39	0.4991	66	35	47	15
1033	8.481	3.676	6.903				
80.08	6.218	12.2	13.79	75	73	92	110
464.3	15.75	9.153	1.489	68	44	59	17
485.7	9.932	7.064	11.26				
269.3	0.86	4.324	11.24	58	34	52	11
351.6	13.18	11.51	8.314	78	45	53	17
2704	10.13	11.93	27.59	92	35	35	11
1622	8.43	6.623	10.76				
3308	0.01588	4.773	1.221	97	34	33	9
3233	0.4721	2.391	3.732				
	9.823	15.34	18.27	97	87	83	85
419.4	15.54	5.761	14.31	78	34	40	12
131.1	10.5	9.711	6.886	88	83	85	79
166.1	7.153	8.357	12.67	93	84	87	83
	19.78	7.942	5.266	46	10	36	4
	14.1	7.656	15.47	51	15	27	6
	7.3	13.42	18.07				
	6.222	3.14	2.301				
	14.24	5.639	5.37				
	9.132	8.175	7.409				
	3.875	5.306	3.228	49	15	28	6
	2.343	6.987	11.84	43	18	39	7
134.5	18.13	65	13.03	67	68	92	92
112.5	3.027	7.025	20.6	97	74	68	42
	7.367	26.73	11.63	66	39	55	16
	5.839	16.37	2.994	69	38	52	14
132.6	7.947	17.48	4.805				
54.19	8.662	54.1	10.68	78	88	100	130
399.6	0.6726	3.414	7.161	82	65	71	39
70.95	5.586	6.617	13.33	80	66	75	43
28.6	18.32	17.47	4.932	124	132	92	173
28.47	7.995	22.35	5.171	124	132	92	173
41.92	15.97	4.586	0.1709	103	108	93	141
73.58	4.054	64.6	25.65	100	105	93	127
181.7	25.68	30.35	21.1				
165.8	23.38	66.81	15.13				
	11.12	21.23	14.64				
	10.42	38.59	7.313				
231.2	7.456	5.381	0.6738				
146.6	9.108	37.3	13.21				

463.8	25.47	3.86	5.691
236.2	15.93	28.26	7.767
93.99	10.68	16.67	5.982
142.5	7.848	30.94	3.806

TLC	RV	RV_TLC	Raw	Raw_p	sRaw	sRaw_p	FVC_gr
91	70	31					0
114	92	18					0
95	145	33	7.39	164	9.33	189	0
96	53	12	2.97	62	3.98	83	0
106	144	30	2.87	64	4.01	81	0
97	99	22	4.39	94	7.64	157	0
77	107	32	2.64	58	3.28	67	1
							0
115	202	43	5.26	89	6.07	143	0
71	94	30	1.7	138	4.74	106	1
108	228	56	13.6	928	58.21	1514	1
							1
							1
79	42	28	2.71	242	5.51	126	1
92	120						0
							1
							1

							1
							0
							1
92	210	49	6.22	165	14.85	280	1
104	204	42	5.26	144	13	243	0
131	265	41	5.05	383	27.07	597	0
136	291	44	5.72	448	31.58	701	0
86	33	33	1.61	70	5.4	90	0
170	434	60	16.34	315	45	980	0
81	59	16	1.93	48	3.42	66	0
							0
							1
							1
133	453	71	17.47	556	83.6	1492	1
115	372	67	15.33	492	61.82	1101	1
							1
							0
88	22	42	5.65	156	11.72	218	1
91	175	42	5.18	141	12	225	1
99	100	42	4.86	88	5.65	127	0
							0
115	245	46	4.48	112	12.5	241	0
110	72	15	3.63	58	4.41	109	0
110	72					109	0
95	72	17	3.63	68	5.01	111	0
95	81	19	3.12	66	4.71	92	0

FEV1_gr	FEV1_FVC_	FEF25_75_	Raw_p_gr	sRaw_p_gr	TLC_gr	FVC_gr1	FEV1_gr1
0	0	0			0	0	0
0	0	0			0	0	0
0	0	0	1	0	0	0	1
0	0	0	0	0	0	0	0
0	0	0	0	0	0	0	0
0	0	0	0	0	0	0	0
1	0	0	0	0	0	1	1
0	0	0				1	0
0	0	0	0	0	0	1	0
1	0	0	1	0	0	1	1
1	1	1	1	1	0	1	2
1	1	1				2	2
1	1	1				2	2
1	0	1	1	0	1	2	2
1	0	1			0	0	1
1	1	1				1	2
1	1	1				2	2

1	1	1				1	2
0	0	0				1	1
1	1	1				1	2
1	1	1	1	1	0	2	2
1	1	1	1	1	0	1	2
1	1	1	1	1	0	0	2
1	1	1	1	1	0	0	2
0	0	0	0	0	0	0	0
1	1	1	1	1	0	1	2
0	0	0	0	0	0	0	0
0	0	0				0	0
1	1	1				2	2
1	1	1				2	2

1	1	1	1	1	0	2	2
1	1	1	1	1	0	2	2
1	0	0				1	1
0	1	1				0	1
1	1	1	1	1	0	1	2
1	1	1	1	1	0	1	2
0	0	0	0	0	0	1	0
1	0	1				0	1
1	0	1	1	1	0	0	1
0	0	0	0	0	0	0	0
0	0	0		0	0	0	0
0	0	0	0	0	0	0	0
0	0	0	0	0	0	0	0

FEV1_FVC_	FEF25_75_	TLC_gr1	DLCO	DLCOadj	VA	DLCO_VA	DLCOadj_
0	0	0	49.38374	52.71439	61.04537	79.70832	88.19653
0	0	0	74.15989	76.6	89.3953	81.86261	87.52557
0	1	0					
0	0	0	60.63558	68.71	88.80823	67.48257	79.4691
0	0	0					
0	0	0					
0	0	1	56.58946	59.5	65.03704	85.97265	90.10271
0	0						
0	0	0					
0	1	1	75.21543	75.22	75.25261	99.53314	99.25434
2	2	0					
2	2						
1	2						
1	2	2	16.79496	17.09	31.85554	52.48434	53.2507
1	2	0					
2	2						
1	2						

2	2						
0	0						
2	2						
2	2	0	58.00038	59.25736	51.24794	112.2081	116.7242
2	2	0	64.17064	326.7512	70.3805	90.39692	477.7411
2	2	0					
2	2	0					
0	0	0	93.85366	88.81293	77.22361	121.1113	116.465
2	2	0					
0	1	0	58.38333	63.01493	86.27173	66.86175	76.20724
0	0						
2	2						
2	2						
2	2	0					
2	2	0					
0	0						
1	2						
2	2	0	43.26015	47.27208	248.3648	17.46084	18.95061
2	2	0	42.5946	43.22664	65.63927	65.05161	70.60185
0	0	0	59.0399	66.33306	85.42738	66.92445	76.29387
1	2						
1	2	0	87.91526	90.93197	65.7311	131.9492	143.7211
0	0	0	101.0445	101.3671	78.92461	126.2288	133.0784
0	0	0	91.64003	92.83794	93.57462	96.3623	102.6605
0	0	0	91.64003	92.83794	93.57462	96.3623	102.6605
0	0	0	81.0253	80.27275	474.2333	16.85335	17.53166

DLCO_mL/	VA_L	Hb
5.5	1.16	11.6
9.3	1.96	12.5
7.5	1.93	10.2
6.4	1.27	12
21.2	4.02	10
5.7	2.15	14.1

9.4	1.5	13.3
10.4	2.06	12.8
30.2	4.79	15.1
8.5	2.25	10.1
6.5	7	11.1
6.4	1.85	11.5
5.7	1.37	10
12.9	1.71	13.9
11.2	1.5	12.7
9	1.53	12.7
9	1.53	12.7
9.3	9.4	13.3
