# Supplementary material for: Impulse oscillometry system for assessing small airway dysfunction in pediatric bronchiolitis obliterans; association with conventional pulmonary function tests
Source: PLoS One. 2023 Feb 7;18(2):e0280309. doi: 10.1371/journal.pone.0280309 (PMC9904454; doi:10.1371/journal.pone.0280309)
Supplement: Attachment — Submitted filename: Response to Reviewers_IOS_1214.docx [file pone.0280309.s002.docx]

**< Response to Reviewers >**

- We sincerely appreciate the reviewers for their comments and questions, and below we address each comment and question in detail.

**#1. The categorical and continuous variables were compared using the χ2 and Student’s t‐ test, respectively -> Please cite the statistical guideline such as DOI: https://doi.org/10.54724/lc.2022.e1**

- The manuscript has been revised on page 5 and Table 1 as follows, according to the statistical guidelines suggested by the reviewers.

: Descriptive analyses of qualitative variables are expressed as number of patients for each category and percentage. Quantitative variables are presented as mean and standard deviation for normally distributed variables or as median and interquartile range (IQR) when not. Comparisons between patients with BO and control group were done using Chi-square test for categorical variables and Mann-Whitney test for continuous variables. If there is at least one cell that has a frequency of five or less, an additional correction was done with Fisher exact test. (page 5)

**#2. IOS and PFT parameters were analyzed using Spearman's rank correlation since they did not follow a normal distribution, and we performed multivariate logistic and linear regression analysis between these two variables
-> If the normal distribution is not satisfied, linear correlation and logical regression models should not be performed.
i.e.) Yon DK, Hwang S, Lee SW, Jee HM, Sheen YH, Kim JH, Lim DH, Han MY. Indoor Exposure and Sensitization to Formaldehyde among Inner-City Children with Increased Risk for Asthma and Rhinitis. Am J Respir Crit Care Med. 2019 Aug 1;200(3):388-393. doi: 10.1164/rccm.201810-1980LE. PMID: 30958969.
TZCA, total eosinophil count, total IgE, Rrs5, and FeNO data were log transformed and Rrs5–20 data were square root transformed to obtain normal distributions.
Please transform to obtain normal distributions (using log- or square root-transformation).**

- The manuscript has been revised on page 5 and Table 2 as follows, in response to the reviewers' comments:

: The IOS data were square-root transformed to obtain a normal distribution (Table 2), and a general linear model analysis between the conventional PFTs was presented with beta coefficients with 95% confidence intervals (CIs). Results of the multivariate logistic regression with obstructive PFT parameters presented as odds ratios (OR) with 95% CI. All multivariate outcomes were adjusted for confounding by age and sex (based on the assumption that these are potential confounders). (page 5)

**Reviewer #1:**

**1. Title – impulse oscillometry cannot be used for the diagnosis of bronchiolitis obliterans. It can be used for the assessment of pulmonary function in patients with bronchiolitis obliterans diagnosed in CT**

- The title has been revised on page (1) as follows, in response to the reviewers' comments:

: “Impulse oscillometry system for assessing small airway dysfunction in pediatric bronchiolitis obliterans; association with conventional pulmonary function tests”

**2. There are published studies on good correlation between resistance indices of impulse oscillometry and spirometry as well as body plethysmography parameters. Why did the authors suspect that it might be different in bronchiolitis obliterans? This should be addressed.**

- The manuscript has been revised on the page (3, 6) as follows, in response to the reviewers' comments:

: The correlation between IOS and conventional PFT has already been evaluated in children with asthma [7,9-12], cystic fibrosis [13,14] and other chronic obstructive pulmonary diseases [15,16]. (page 3). To the best of our knowledge, studies of IOS with pediatric BO patients are limited. In young children with BO, irreversible fixed small airway obliteration occurs in a more severe form compared to other obstructive lung diseases. Therefore, spirometry requiring forced ventilation or the body box with long test times have a low success rate, particularly in severe cases. (page 3)

: In this study, out of 89 IOS measurements, only 43 spirometry and 29 body plethysmographic measurements were performed simultaneously, and there were cases of failure in patients who were too young or unable to perform forced breathing due to severe BO. (page 6)

: The purpose of this study is, first, to verify the correlation between IOS, spirometry, and body plethysmography in children with BO. Second, and more importantly, we tried to suggest that the IOS parameters are relatively easily obtainable, and reliable indicators even in patients with severe BO who cannot perform a conventional PFTs. (page 3)

**3. Study population: the author state that in the control group was 1 healthy child. Yet later this healthy child is described as having Swyer James syndrome which can definitely influence the breathing mechanics. I do not think this child should be included in the control group**

: The 15 patients included in the control group of our study were 14 patients without previous lung disease who underwent pre-transplant pulmonary function evaluation in Dep. of hemato-oncology, and 1 healthy child who underwent pulmonary function evaluation for a regular health checkup.

: The SJS patients indicated by the reviewers were included in the BO group, not the control group (Table 1). We have described this in the study population section as follows

: Patients who underwent IOS with (b) pre-transplant evaluation in Department of hemato-oncology without previous lung disease (n=14), and (c) healthy child (n=1); Both (b) and (c) were included in the control group. (page 4)

**4. Results: Author report that “R5 values were significantly worse in the BO group” yet in table 1 no such difference is present for raw R5 values P=0.786. This leads to another issue: why does the Author present the results differently: sometimes as raw and predicted values (table 1) and sometimes as z-scores (table 2 and 3) this should be explained**

- The authors agree that it is necessary to clarify the specific unit of the IOS parameter in the results section. The manuscript has been revised in result section on page 6 and discussion on page 11 as follows, in response to the reviewers' comments:

: Most of the IOS parameters, R5-Z, R5 %, R5-20, X5, AX, AX-Z, AX % were statistically significantly worse in the BO group (Table 1, Fig 1) (page 6)

: In the BO group, most of the IOS parameters were significantly worse except raw R5 cmH2O/L/s. As there is no general reference range of IOS specific to pediatric patients to date, we assumed that validation should include raw values, Z-scores, and % predictive values with all different units. (page 11)

**5. Linear Correlation – Raw is not a spirometry parameter**

- The manuscript has been revised the title of table 2 (on page 8) as follows, in response to the reviewers' comments:

: General linear regression analysis of airway resistance and reactance parameters between IOS, spirometry, and plethysmographic resistance.

**6. ROC and Logistic regression: Why did the Author choose 103% for Raw and 203% for sRaw as cut-off values. If the aim of the study was to compare both IOS and body plethysmography parameters with spirometry it should have been stated in the aims**

- The manuscript has been revised in the introduction section on the page (3) as follows, in response to the reviewers' comments:

: In young children with BO, irreversible fibrosis and fixed small airway obliteration occurs in a more severe form compared to other obstructive lung diseases. Therefore, spirometry requiring forced ventilation or the body box with long test times has a low success rate, particularly in severe cases. The purpose of this study is, first, to verify the correlation between IOS, spirometry, and body plethysmography in children with BO. Second, and more importantly, we tried to suggest that the IOS parameters are relatively easily obtainable, and reliable indicators even in patients with severe BO who cannot perform a conventional PFTs. (page 3)

: The normal reference ranges for sRaw also have not been established in children, so we used 203.5% as the cut-off values following our ROC analysis in this study. (page 7)

**7. ROC and Logistic regression: Why did the author chose as the criteria of obstructive patterns FEV1, FEV1/FVC (I presume %predicted- not stated in the text) of 75 and FEF25075% of 60? The spirometric criterium of obstructive pattern of ventilation is FEV1/FVC z-score <-1.645. The author also does not state whether just 1 or all the three criteria had to be fulfilled.**

- The manuscript has been revised on the page (7-8) as follows, in response to the reviewers' comments:

**:** The cutoffs for normal values used for FEV1, FEF25-75% and sRaw were 75%, 60% and 203.5% of predicted, respectively. The obstructive group was defined when one of these criteria was met. We used a cutoff value for FEV1 < 75% based on the criteria for the definition of BO from the National Institutes of Health (NIH) consensus, 2014 [20]. FEF25-75% is a potentially sensitive parameter for evaluating small airway function in pediatrics [21]. There are no guidelines regarding normal values in children, and previous studies have suggested thresholds of 60% or 65% in children to define peripheral airflow obstruction [22,23].

**8. DISCUSSION: what does: The deterioration of the peripheral airway tissue” mean?**

- The manuscript has been revised on the page (12) as follows, to help better understand the context of the manuscript.

: Loss of compliance due to extensive fibrosis of peripheral airway tissue in BO patients can be better represented by this overall value than by resistance or reactance parameters at specific frequencies.

**9. All the abbreviations used in tables should be explained**

- We added the abbreviations in Tables 1-3 to help better understand the manuscript.
